# Assessment of quality and reliability of YouTube videos for patient and physician education on inflammatory myositis

**DOI:** 10.1007/s10067-023-06522-x

**Published:** 2023-02-09

**Authors:** Mrudula Joshi, Naveen R., Kshitij Jagtap, Ria Gupta, Vikas Agarwal, Rohit Aggarwal, Ashish Goel, Latika Gupta

**Affiliations:** 1 Byramjee Jeejeebhoy Government Medical College and Sassoon General Hospitals, Pune, India; 2 Department of Clinical Immunology and Rheumatology, Sanjay Gandhi Postgraduate Institute of Medical Sciences, Lucknow, India; 3 Seth Gordhandas Sunderdas Medical College and King Edward Memorial Hospital, Maharashtra Mumbai, India; 4 Department of Medicine, Rheumatology and Clinical Immunology, University of Pittsburgh, Pittsburgh, PA USA; 5 Department of Medicine, Dr BR Ambedkar State Institute of Medical Sciences, Mohali, Punjab India; 6 Department of Rheumatology, Royal Wolverhampton Hospitals NHS Trust, Wolverhampton, WV10 0QP UK; 7 Division of Musculoskeletal and Dermatological Sciences, School of Biological Sciences, Faculty of Biology, Medicine and Health, Centre for Musculoskeletal Research, Manchester Academic Health Science Centre, The University of Manchester, Manchester, UK; 8 City Hospital, Sandwell and West Birmingham Hospitals NHS Trust, Birmingham, UK

**Keywords:** Misinformation, Myositis, Patients, Social Media, YouTube

## Abstract

**Introduction:**

YouTube is the second most popular search website worldwide to access health information online. This study was undertaken to assess the reliability and quality of information about myositis on YouTube and delineate attributes of useful videos using standard metrics.

**Methods:**

We conducted a thorough search on YouTube using 9 search terms related to myositis. The inclusion criteria were content related to myositis, English language and acceptable audio–video quality. Duplicates and advertisements were excluded from the analysis. Videos were classified as useful, not very useful or misleading and patient narratives. Reliability was determined using the mDISCERN criteria, quality using the Global Quality Scale (GQS) and JAMA system, using appropriate cut-offs (mDISCERN > 4, GQS > 4, JAMA > 3).

**Results:**

Out of a total of 900 videos, 453 were included for the analysis. Seventy-four per cent and 2% provided useful and not very useful information respectively, while 24% were patient narratives. Seventy-one per cent were intended specifically for patients while 69% were for healthcare providers and students.

Noteworthily, useful and not very useful videos had similar total views though the number of likes and daily viewership were higher for useful videos (*p* = 0.024, *p* = 0.046).

Nearly half (47%) of useful videos were by professional medical societies/patient support groups (PSGs) while not very useful ones were by nonmedical media (38%). Physician-predicted usefulness was discordant with score-based usefulness (*κ* = 0.129). However, GQS emerged as a significant (*p* = 0.008) predictor of video usefulness in multivariate analysis.

**Conclusion:**

A large majority of English YouTube videos on myositis provide useful information for patients. Physicians could signpost patients to high-quality useful videos as determined by GQS and sources like professional medical societies and PSGs.

**Table:** 

**Key Points** *•This study highlights the importance of regulating health information posted online, accessed by millions of people, to gauge the quality of information and to identify and curb misinformation.* *•It also identifies recommendations for the future for uploading such content on the Internet.* *•The implications lie in our patients being better informed about their disease as they are important stakeholders in the healthcare decision-making process.*

**Supplementary Information:**

The online version contains supplementary material available at 10.1007/s10067-023-06522-x.

## Introduction 

In the digital era that we live, eight of ten users access health information on the internet [1]. Of the various sources, YouTube is the second most popular search platform worldwide [1]. It is also one of the most visited platforms for seeking health-related information, especially for rare disorders of health. Abundant information about different health-related events is available on the platform. Its audio-visual interface makes it easier to register and retain information. Although the information on YouTube is subjected to a stringent copyright check, the same cannot be said about their reliability and quality-check process. The misinformation on the internet makes it imperative to secure an understanding of the quality of health-related disease-specific information online. Recent quality assessment studies on rheumatic diseases like systemic lupus erythematosus, rheumatoid arthritis and gout have identified content that may not be useful and even misleading at times [2–4]. As more and more patients increasingly turn to the Internet to seek health information online, it becomes pertinent for healthcare providers to identify platforms or sources providing reliable and high-quality information and to assist patients in evaluating the same [5, 6].

The idiopathic inflammatory myopathies are not only rare but also complex conditions, which are often associated with comorbidities and complications of prolonged treatment [7]. A typical consultation for myositis or another complex connective tissue disease requires several aspects to be addressed and presumably takes longer than other general medical consults. Often, patients feel overwhelmed with the information that is provided to them in a short interview, leaving gaps in understanding resulting in poor drug compliance. This opens an area of felt need for additional information support which patients seek with the help of online resources. Identifying the determinants of quality videos can help time-constrained physicians to guide patients to appropriate, reliable, accessible and understandable information.

We undertook a cross-sectional review of the reliability and quality of YouTube videos on myositis using validated assessment tools (modified DISCERN criteria, Global Quality Scale (GQS) and the JAMA scoring system) [3, 8, 9]. We triangulated baseline features, views and usage data with video utility using predefined criteria to identify the determinants of useful videos. Sources of useful information were identified.

The results of this study will help determine the percentage of misinformation present about myositis on YouTube and identify the type, distribution and quality of content present, which will help physicians to direct patients to useful videos. Knowing the type and quality of content present will help plan future strategies for creating videos for supplementing patient and physician education, which may include the production of videos on the lesser prevalent content, and the debut of renowned healthcare providers on YouTube.

## Methodology

In a cross-sectional design, we evaluated the quality of videos on YouTube® related to myositis after a thorough search using terms “Myositis”, “Idiopathic Inflammatory Myositis”, “Dermatomyositis”, “Polymyositis”, “Cancer Associated Myositis”, “Inclusion Body Myositis”, “Immune Mediated Necrotizing Myopathy”, “Juvenile Dermatomyositis” and “Overlap Idiopathic Inflammatory Myositis” sequentially in March 2021. Search terms were determined based on the type and classification of myositis. The internet browser was cleared of cookies and search history prior to the search which was carried out in the incognito mode to avoid bias from previous searches.

The first ten pages, i.e. one-hundred videos per search term, were included in the evaluation, as viewers rarely go beyond this. We searched videos using the ‘relevance’ filter, which is the default setting on YouTube®, thus duplicating the search results of the common population and providing accurate results. The first 100 videos for each term were saved to a playlist for future analysis. Two authors (MJ and RG) conducted the search and evaluated the videos independently while LG resolved conflicts if any.

### Inclusion and exclusion criteria

**Fig. 1:**
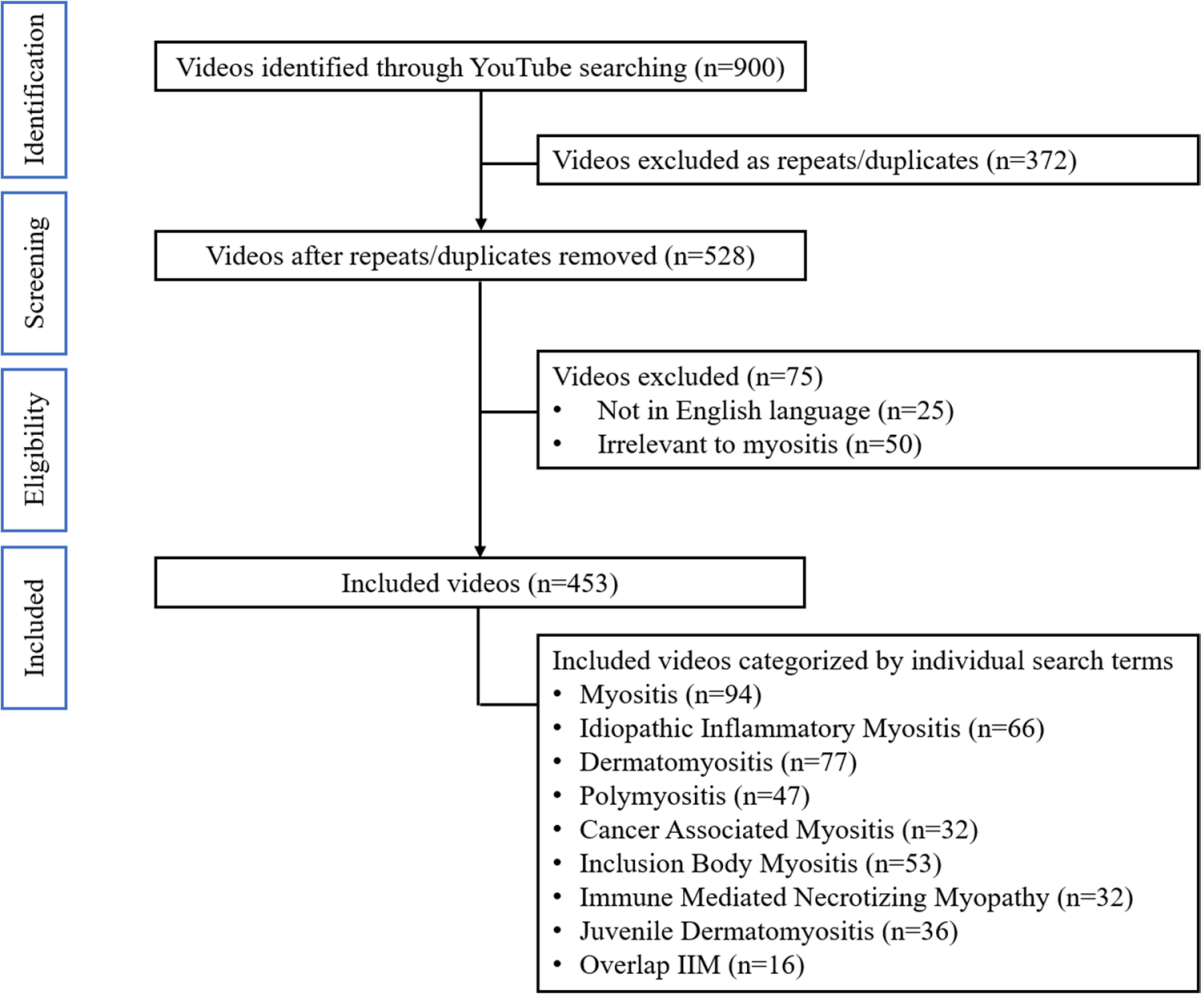
Flowchart of selection of YouTube® videos for the study

We included videos that had primary content related to myositis, in English language, with acceptable audio–video quality, and were available on 13 March 2021. Multi-part videos were evaluated as a single entity. We excluded videos that did not relate to myositis, were repeats or duplicate or were advertisements. Of the 900 videos initially screened, 453 videos were included. The details are presented in Fig. 1.

### Video characteristics

The videos were classified as useful, not very useful or misleading, or patient narratives by two independent assessors, as defined in Table 1 [2]. A third independent assessor resolved bias, if any. A total of 21 video characteristics were collected including title, channel name, number of subscribers, category (treatment, aetiology, diagnosis, signs and symptoms, ancillary care, diet, physiotherapy, adverse drug reactions, physical examination, risk factors, pathogenesis, patient experience and miscellaneous), upload date, duration, number of views, days since upload, viewing rate, daily viewership, number of likes, number of dislikes, interaction index, source (hospital, group practice or physician, non-medical independent user, non-medical media organization, professional medical body/patient support group OR pharmaceutical company) (Online Resource 1), hashtag use, age (> 18 or all), number of comments, intended audience (anyone/general public, specifically for patients, healthcare providers including students and caregivers), YouTube ID/channel URL, image quality (poor, good and high), training level of the speaker (formal medical training and no formal medical training) and speciality of the speaker (if doctor-rheumatologist, dermatologist, neurologist, general medicine physician and others/unknown).

**Table 1:** Video content evaluation [2]

Video category	Definition
*Assessment of usefulness*	
Useful videos	Videos mainly focusing on delivery of information and containing clinically accurate information on any aspect of the disease and were useful for learning about myositis
Not very useful videos/misleading	Videos containing clinically incorrect or unproven information based on currently available scientific evidence. Videos containing partially useful and partially misleading information were also classified as not very useful
Patient narratives	Videos describing a patient’s personal experiences and/or feelings about myositis which provided emotional support to the audience
*Assessment of reliability: modified DISCERN tool*	
1	Is the video clear, concise and understandable?
2	Are valid sources cited? (From valid studies, physiatrists, or rheumatologists)
3	Is the information provided balanced and unbiased?
4	Are additional sources of information listed for patient reference
5	Does the video address areas of controversy/uncertainty?
*Assessment of quality: Global Quality Scale (GQS)*	
1	Poor quality, poor flow of the video, most information missing, not at all useful for patients
2	Generally poor quality and poor flow, some information listed but many important topics missing, of very limited use to patients
3	Moderate quality, suboptimal flow, some important information is adequately discussed but others poorly discussed, somewhat useful for patients
4	Good quality and generally good flow. Most of the relevant information is listed, but some topics not covered, useful for patients
5	Excellent quality and flow, very useful for patients
*Assessment of quality: JAMA scoring system*	
Authorship	Authors and contributors, their affiliations, and relevant credentials should be provided
Attribution	References and sources for all content should be listed clearly, and all relevant copyright information should be noted
Disclosure	Website “ownership” should be prominently and fully disclosed, as should any sponsorship, advertising, underwriting, commercial funding arrangements or support, or potential conflicts of interest
Currency	Dates when content was posted and updated should be indicated

A *daily viewership rate* was calculated in order to avoid bias that may result from the date of upload to YouTube®, using the formula: total view count/duration of availability. The total view count was noted during viewing of the video. The duration of availability was calculated by subtracting the date of upload from the date of viewing in days. Interaction index and viewing rate were calculated using the following: (number of likes-number of dislikes)/total number of views × 100% and (number of views/number of days since upload × 100%) respectively.

### Video content evaluation

The reliability of the videos was determined using the modified DISCERN criteria, a scale for assessment of audio–video content (Table 1) [3]. It is a 5-point scale where each element is scored individually. Quality of the videos was determined by the GQS (Table 1) [8]. A higher score on both the scales implies greater reliability and quality respectively. Additionally, the JAMA scoring system was also used to determine the quality of videos (Table 1) [9]. It is a 4-point scale, in which videos are scored on the basis of 4 parameters, namely authorship, attribution, disclosure and currency. Similar to GQS, a higher score on this scale implies greater quality of the video.

### Statistical analysis

Continuous variables are presented as mean (± standard deviation) or median (interquartile range) depending upon normality of data, and categorical variables are presented as counts and percentages. The results were analysed using the nonparametric Kruskal–Wallis test and the Pearson chi-squared test for continuous and categorical variables respectively. A *p*-value of < 0.05 was considered significant. Inter-rater reliability was evaluated for mDISCERN, JAMA and GQS scores using intraclass correlation (ICC) estimates and their 95% confidence intervals based on a mean-rating (*κ* = 3), absolute-agreement, two-way mixed-effects model.

### Ethical considerations

No human subjects were involved in the conduct of this study. The study included a review of video content posted on YouTube® which falls under the category of open source distribution. The study was exempt from ethical review.

## Results

A total of 900 videos were screened for inclusion in the study. Of these 372 duplicates, 25 videos presented in a language other than English and 50 that were considered irrelevant were excluded. A total of 453 videos were identified for further analysis. After detailed viewing, 108 were found to be patient narratives and experiences, and eight were identified as not very useful for patients and doctors.

### Video characteristics

Of the analysed content, 191 (42%) videos were uploaded by a professional medical society or a patient support group (PSG). Furthermore, 79 (17%) were developed by group practice, physician and 62 (14%) by a hospital. While reviewing the videos, it was observed that 316 (70%) were presented by a speaker with formal medical training which included rheumatologists (16%) and neurologists (12%).

### Audience reception and Viewership

The median number of views was 985 (IQR, 339–3388) and the daily viewership was found to be 139 (IQR, (− 412 to 2069). The detailed baseline characteristics of the videos are presented in Table 2. While there was no statistically significant difference in the median views between the useful and not very useful videos, the median number of likes (*p* = 0.02) and the median number of views per day (*p* = 0046) or daily viewership were significantly higher for useful videos. The interaction index for useful videos was significantly higher (*p* = 0.01).

**Table 2:** Baseline characteristics of YouTube videos

Video characteristics	Useful (*N* = 337)	Not very useful (*N* = 8)	Patient narratives (*N* = 108)	Total (*N* = 453)
*Audience interaction parameters, median (IQR)*				
No. of views	921 (275–3296)	412.5 (146–2599)	1462 (608–3631)	985 (339–3388)
Subscribers	4110 (784–6330)	5635 (1162–172,385)	664 (131–4110)	3500 (370–5555)
Days since upload	912 (463–1616)	1436.5 (884–2119)	982 (476–2179)	954 (472–1647)
Viewing rate	115 (45–304)	31 (10–335)	150 (76–375)	126 (49–325)
Number of likes	12 (4–34)	1 (0–50)	25 (12–46)	14 (5–36)
Number of dislikes	0 (0–2)	0 (0–2)	0 (0–1)	0 (0–1)
Interaction index	1.07 (1–2)	0.16 (0–1)	1.27 (1–3)	1 (1–2)
Number of comments	0 (0–3)	0 (0–4)	2 (0–7)	1 (0–4)
Daily viewership	95 [(− 459) to 2001]	− 1136 [(− 1587) to 1833]	396.5 [(− 145) to 2190]	139 [(− 412) to 2069]
*Intended audience, n (%)*				
Anyone/general public	17 (5)	2 (25)	5 (5)	24 (5)
Specifically for patients	212 (63)	7 (88)	105 (97)	324 (71)
Healthcare providers including students	304 (90)	4 (50)	5 (5)	313 (69)
Caregivers	51 (15)	2 (25)	22 (20)	75 (17)
*Content category, n (%)*				
Treatment	136 (40)	3 (37)	2 (2)	143 (32)
Aetiology	30 (9)	0 (0)	0 (0)	30 (7)
Diagnosis	136 (40)	1 (12)	0 (0)	137 (30)
Signs and symptoms	123 (37)	2 (25)	0 (0)	125 (28)
Ancillary care	6 (2)	1 (13)	0 (0)	8 (2)
Diet	8 (2)	0 (0)	0 (0)	8 (2)
Physiotherapy	20 (6)	0 (0)	1 (1)	22 (5)
ADRs	7 (2)	0 (0)	0 (0)	7 (1.5)
Physical examination	6 (1)	0 (0)	0 (0)	7 (1.5)
Risk factors	9 (3)	0 (0)	0 (0)	9 (2)
Pathogenesis	47 (14)	0 (0)	0 (0)	47 (10)
Patient experience	4 (1)	0 (0)	99 (92)	103 (23)
Miscellaneous	119 (35)	4 (50)	12 (11)	138 (31)
*Sources of upload, n (%)*				
Hospital	53 (16)	1 (13)	8 (7)	62 (14)
Group practice or physician	74 (22)	1 (13)	4 (4)	79 (17)
Nonmedical independent user	14 (4)	0 (0)	40 (37)	54 (12)
Nonmedical media organization	27 (8)	3 (38)	7 (6)	37 (8)
Professional society/support group	158 (47)	3 (38)	30 (28)	191 (42)
Pharmaceutical company	11 (3)	0 (0)	19 (18)	30 (7)
*Training level of the speaker, n (%)*				
Formal medical training	310 (92)	4 (50)	2 (2)	316 (70)
No formal medical training	27 (8)	4 (50)	106 (98)	137 (30)
*Speciality of speaker, n (%)*				
Rheumatologist	74 (22)	0 (0)	0 (0)	74 (16)
Dermatologist	13 (4)	0 (0)	0 (0)	13 (3)
Neurologist	55 (16)	1 (13)	0 (0)	56 (12)
GP	11 (3)	0 (0)	1 (1)	12 (3)
Others/unknown	154 (46)	3 (38)	1 (1)	158 (35)

*ADRs* adverse drug reactions, *GP* general physician, *GQS* Global Quality Scale

### Target audience

Among the included videos, 324 (71%) were developed for a target audience of patients, 313 (69%) for healthcare providers including students and 75 (17%) for caregivers. Videos contained information on the treatment (143, 32%), diagnosis of myositis (137, 30%), signs and symptoms (125, 28%) and miscellaneous information (138, 31%).

### Video usefulness and reliability

Of the analysed videos, 74.3% (337/453) provided information that was considered useful and 1.7% (8/453) not very useful. It was found that content uploaded by non-medical media organizations was found not very useful videos (3 of 8 videos, 38%). Content created by professional medical societies/patient support groups was considered useful (158 of 337; 47%). Content created by group practice or physician was also considered useful (74 of 337; 22%). As many as 310 (92%) videos were created by professionals who had undergone formal medical training. The detailed baseline characteristics of the videos, stratified by the source, are presented in Online Resource 2.

#### Reliability

The *reliability* of the videos as assessed by the modified DISCERN criteria was higher for useful videos as compared to the not very useful videos (4.0, range 3.0–4.0 vs 2.0, range 1.0–3.0), with a significant statistical difference (*p* ≤ 0.001). The useful videos also had higher scores for quality obtained by GQS (4.5, range 3.5–5.0 vs 1.0, range 1.0–2.8) and JAMA (3.0, range 3.0–4.0 vs 2.2, range 2.0–3.0) compared to the not very useful videos, and this was statistically significant (*p* ≤ 0.001 and *p* = 0.004, respectively). A comprehensive analysis of video characteristics by usefulness category is given in Table 3.

**Table 3:** Analyses of video characteristics by usefulness category

Video characteristics	Useful (*N* = 337)	Not very useful (*N* = 8)	OR (CI)	*P* value
*Audience interaction parameters*				
No. of views	921 (275–3296)	412.5 (146–2599)		0.274
Subscribers	4110 (784–6330)	5635 (1162–172,385)		0.274
Days since upload	912 (463–1616)	1436.5 (884–2119)		0.177
Viewing rate	115 (45–304)	31 (10–335)		0.104
Number of likes	**12 (4–34)**	**1 (0–50)**		0.024
Number of dislikes	0 (0–2)	0 (0–2)		0.727
Interaction index	**1.07 (1–2)**	**0.16 (0–1)**		0.012
Number of comments	0 (0–3)	0 (0–4)		0.543
Daily viewership	**95 [(− 459) to 2001]**	** − 1136 [(− 1587) to 1833]**		0.046
*Intended audience, n (%)*				
Anyone/general public	**17 (5)**	**2 (25)**	6.2 (1.2–33)	0.014
Specifically for patients	212 (63)	7 (88)		0.153
Healthcare providers including students	**304 (90)**	**4 (50)**	0.1 (0.02–0.45)	< 0.001
Caregivers	51 (15)	2 (25)		0.444
*Content category, n (%)*				
Treatment	136 (40)	3 (37)		0.871
Aetiology	30 (9)	0 (0)		0.377
Diagnosis	136 (40)	1 (12)		0.112
Signs and symptoms	123 (37)	2 (25)		0.504
Ancillary care	6 (2)	1 (13)		0.153
Diet	8 (2)	0 (0)		0.659
Physiotherapy	20 (6)	0 (0)		0.478
ADRs	7 (2)	0 (0)		0.680
Physical examination	6 (1)	0 (0)		0.703
Risk factors	9 (3)	0 (0)		0.640
Pathogenesis	47 (14)	0 (0)		0.256
Patient experience	4 (1)	0 (0)		0.757
Miscellaneous	119 (35)	4 (50)		0.423
*Sources of upload, n (%)*				
Hospital	53 (16)	1 (13)		0.804
Group practice or Physician	74 (22)	1 (13)		0.529
Nonmedical independent user	14 (4)	0 (0)		–
Nonmedical media organization	**27 (8)**	**3 (38)**	0.12 (0.03–0.6)	0.018
Professional society/support group	158 (47)	3 (38)		0.601
Pharmaceutical company	11 (3)	0 (0)		–
*Training level of the speaker, n (%)*				
Formal medical training	**310 (92)**	**4 (50)**	11.5 (2.7–48)	< 0.001
No formal medical training	**27 (8)**	**4 (50)**		< 0.001
*Speciality of speaker, n (%)*				
Rheumatologist	74 (22)	0 (0)		–
Dermatologist	13 (4)	0 (0)		–
Neurologist	55 (16)	1 (13)		0.773
GP	11 (3)	0 (0)		–
Others/Unknown	154 (46)	3 (38)		0.646
*Video quality metrics*				
mDISCERN	**4 (3–4)**	**2 (1–3)**		< 0.001
GQS	**4.5 (3.5–5)**	**1 (1–2.8)**		< 0.001
JAMA	**3 (3–4)**	**2.25 (2–3)**		0.004

*p*-value is considered significant at a level of < 0.05. A median (IQR) for scale variables is presented. Chi-square for categorical variables and Mann Whitney test for scale variables

*ADRs* adverse drug reactions, *GP* general physician, *GQS* Global Quality Scale

Numbers highlighted in bold are statistically significant values

The useful videos had higher scores for reliability and quality obtained by modified DISCERN (4.0, range 3.0–4.0 vs 2.0, range 2.0–3.0), GQS (4.5, range 4.0–5.0 vs 3.0, range 3.0–3.5) JAMA (3.0, range 3.0–4.0 vs 2.0, range 2.0–2.5) compared to the misleading videos, and this was statistically significant (*p* < 0.001 for all the three scoring systems). A detailed analysis of video characteristics by score based usefulness is given in Online Resource 3.

#### Agreement

The cut-off for a useful video was more than or equal to 4 for the modified DISCERN criteria and GQS and more than or equal to 3 for the JAMA scoring system. Of the analysed videos, 67.1% (304/453) were categorized as useful and 9.0% (41/453) as not very useful videos, based on the aforementioned criteria. The Kappa values for *agreement* between modified DISCERN ≥ 4, GQS ≥ 4, JAMA ≥ 3 and physician decided usefulness were 0.061, 0.083 and 0.104 respectively. The combined Kappa value for agreement between score-based usefulness and physician-decided usefulness was 0.129. The Cohen’s Kappa statistics demonstrating inter-observer agreement were 0.686, 0.469 and 0.537 for mDISCERN, GQS and JAMA scores respectively.

#### Predicting video usefulness

In order to identify the features of a video with useful content, we performed a multivariable binary logistic regression analysis. It was noted that the GQS scoring system is a statistically significant factor (*p* = 0.002) for predicting video usefulness.

## Discussion

The present study explored characteristics, reliability and the quality of the video content on myositis available on YouTube and found that three-quarters of the videos were produced by trained medical professionals. We evaluated a total of 453 videos, with a median of 985 views that addressed different aspects of the disease including signs and symptoms, diagnosis and treatment options. The videos on YouTube had a median viewing rate of 126 views per day indicating that audience frequently accesses content on this platform to gain knowledge on myositis.

Among the top YouTube channels producing content for myositis were Myositis Association, Myositis Support and Understanding, Myositis, Cure JM Foundation and Johns Hopkins Rheumatology. Myositis being a rare, underdiagnosed and heterogeneous group of systemic autoimmune disease, poorly understood by generalist doctors, a felt need for an acceptable, readily available, valid content developed by a reliable source is identified. The significant difference identified between the quality of videos developed by medical groups indicates a need for specialists to develop valid and reliable content [10].

Majority of the videos were targeted toward patients (63%) and students (90%). Interestingly, the median views on the videos identified by our reviewers as useful were not significantly different from the not very useful ones. Indeed, those identified “not very useful videos” had higher number of median subscriptions as compared to “useful ones”. Most of the not very useful videos were produced by nonmedical media organizations and professional society/support groups, with patients being the primary intended audience. However, the quality and flow of the videos were generally poor, with many essential topics missing. One may postulate that the viewers can relate more closely and identify with the presenter in the videos developed by nonmedical individuals, making them more likely to subscribe to their channel. The disparity in the number of subscribers might also be because of the evident difference in the number of useful and not very useful videos. However, this might allow the spread of misinformation. This makes it important for health workers to direct patients towards appropriate content on the platform during clinical consults.

As anticipated, non-medical organizations uploaded the highest proportion of not very useful videos (3/37) as per physician-determined usefulness. The score-based usefulness on the other hand stated that the highest number of not very useful videos came from “group practice/physician” (54%, 22) compared to only 13% (*n* = 1) as per physician-decided usefulness. This disagreement between physician-decided and score-based usefulness (Kappa value 0.129) could be attributed to physician bias or objectivity in score-based systems. Although, poor agreement between the kappa values of mDISCERN, GQS, JAMA and physician-based usefulness (0.061, 0.083 and 0.104) is likely due to the mutually exclusive nature of the scores judging different aspects of the videos, objective scores should be applied to all videos providing health care-related information. The GQS system, which takes into account the quality and flow of the video and the extent of relevant information covered, was found to better predict physician-identified video usefulness using a binary logistic regression analysis.

In the long run, accreditation metrics for validity and guidelines related to the uploading of factually correct and scientifically backed information may be needed. Other factors related to video characteristics such as duration, upload date, audio–video quality and credentials of the speaker also tend to affect people’s decision of choosing which videos to watch. Moreover, YouTube records a view when a user watches at least 30 s of a video. An average person takes longer than the above-mentioned time period to make a decision about the usefulness of a video. As expected, the videos classified as useful captured a higher number of *likes* (useful-12: not very useful-1) and daily viewership (useful 95: not very useful (− 1136)), an indirect indicator of viewers’ acknowledgement of the quality.

While YouTube has become the second most popular social network and the third most trustworthy source of health-related information after physicians and government healthcare institutions, a further rise in usage of social media platforms is expected in the near future, especially in seeking health-related information [1, 11, 12]. Unfortunately, the ease of use also includes a potential for misinformation. Moving forward, development of an algorithm to predict usefulness of videos with badges to identify usefulness and signify verified information on the platform may be the way to promote scientific and reliable information and curb misinformation on platforms such as YouTube.

While several authors have found social media to be a source of misinformation like in COVID-19, rheumatoid arthritis, vaccines or drugs, we found that such was not the case with myositis, possibly owing to it being a niche area [3, 13, 14]. Different surveys have found the proportion of misinformation from 8.6% in Sjögren’s syndrome, 11.5% in SLE, 12.28% in gout, 14% in spondyloarthritis, 17.4% in systemic sclerosis, 27.5% in methotrexate self-injection education (2016), 30% in rheumatoid arthritis, 50% in self-administer subcutaneous anti-tumour necrosis factor agent injections, 57.3% in methotrexate self-injection education (2022) to 67% in COVID-19 [2–4, 13, 15–20] (Table 4). This is understandable because myositis enjoys a niche area with a complicated pathophysiology.

**Table 4:** Misinformation present on social media platforms

Study	Platform	Misinformation/low-quality videos (%)	Number of videos analysed/respondents	Authors
Myositis (current study)	YouTube	9.05	453	Present study
Autoinflammatory diseases (Nov 2022)	YouTube	64.8	105	Sasse et al. [21]
Systemic sclerosis (Aug 2022)	YouTube	17.4	115	Unal-Ulutatar and Ulutatar [17]
Musculoskeletal ultrasound (Aug 2022)	YouTube	–	58	Cuzdan and Turk [22]
Methotrexate self-injection (Aug 2022)	YouTube	57.3	75	Wilson et al. [18]
Fibromyalgia (Jul 2022)	YouTube	–	200	Macedo et al. [23]
Behçet’s disease (Dec 2021)	YouTube	56	50	Karakoyun and Yildirim [24]
COVID-19 vaccination in rheumatic diseases (Dec 2021)	YouTube	12.51	56	Kocyigit and Akyol [25]
Musculoskeletal ultrasound (Oct 2021)	YouTube	40.1	147	Zengin and Onder [26]
Psoriatic arthritis (Sep 2021)	YouTube	14.8	155	Onder and Zengin [27]
Gout (Jul 2021)	YouTube	12.28	114	Onder and Zengin [4]
Spondyloarthritis (Apr 2021)	YouTube	14	200	Elangovan et al. [16]
Fibromyalgia (Feb 2021)	YouTube	–	102	Ozsoy-Unubol and Alanbay-Yagci [28]
Vaccines (Jan 2021)	Twitter	43	–	Suarez-Lledo et al. [14]
Smoking products (Jan 2021)	Twitter	87	–	Suarez-Lledo et al. [14]
Side effects of biologic therapy (Dec 2020)	YouTube	36.4	75	Zengin and Onder [29]
COVID-19 and rheumatic disease link (Jul 2020)	YouTube	36.9	46	Kocyigit et al. [30]
COVID-19 (Jul 2020)	Multiple social media platforms	67.2	128 (respondents)	Gupta et al. [13]
SLE (Feb 2020)	YouTube	11.5% + 4.9% = 16.4	183	Ng CH et al. [2]
Secukinumab (Jul 2019)	YouTube	34	53	Kocyigit and Akaltun [31]
Ankylosing spondylitis exercises (Jun 2019)	YouTube	33.9	56	Kocyigit et al. [32]
Self-administer subcutaneous anti-tumour necrosis factor agent injections (Jul 2018)	YouTube	50	142	Tolu et al. [20]
Sjögren’s syndrome (Apr 2016)	YouTube	8.6	70	Delli et al. [15]
Methotrexate self-injection (May 2016)	YouTube	27.5	51	Rittberg et al. [19]
Rheumatoid arthritis (2012)	YouTube	30.4	102	Singh et al. [3]

Like many other, our study is also bound by certain limitations. Using a cross-sectional study design, we were unable to capture the dynamicity of a social media platform which gets updated continuously. Findings of other investigators may be at variance due to this dynamicity. Moreover, we only evaluated videos with English content. Evaluating other languages was beyond the scope of our current resources. Additionally, we included only the first one-hundred search results on a single social media platform. A broader inclusion of content as well as platforms is likely to reveal more comprehensive picture. However, we have reason to believe that our results are reasonably representative of a larger body of content across different platforms, and physicians should befriend social media while empowering patients to identify valid, reliable and effective content. Our study systematically evaluated video content available on YouTube and provides a reliable tool to predict usefulness for clinicians and patients. Including videos in languages other than English, reviewing the content available on other social media platforms and understanding the psychological and motivational factors influencing patients’ decision to subscribe to not very useful videos possibly by a thematic analysis may be the direction for future studies.

The present study is one of its kind to study characteristics of information on sub-specialisms like myositis available on YouTube and report that the content is reliable with minimal misinformation. Nearly three quarters of the videos are from verifiable sources, and the results are quite reassuring. While it is challenging to eliminate misleading information easily available online on the internet, the present study also concludes that GQS system is a significant marker to identify reliable, valid and useful content on YouTube for myositis and possibly other medical subjects. Specialists should actively participate in the development of medically related videos using validated tools to disseminate appropriate health information.

## Supplementary Information

Below is the link to the electronic supplementary material.

Supplementary file1 (PDF 31 KB)

Supplementary file2 (PDF 36 KB)

Supplementary file3 (PDF 40 KB)

## Data Availability

The datasets generated during and/or analysed during the current study are available from the corresponding author on reasonable request.
