# Supplementary material for: Assessment of quality and reliability of YouTube videos for patient and physician education on inflammatory myositis
Source: Clin Rheumatol. 2023 Feb 9;42(5):1339–49. doi: 10.1007/s10067-023-06522-x (PMC9910767; doi:10.1007/s10067-023-06522-x)
Supplement: Supplementary file 1 — Supplementary file1 (PDF 31 KB) [file 10067_2023_6522_MOESM1_ESM.pdf]

Assessment of Quality and Reliability of YouTube Videos for Patient and Physician Education on Inflammatory Myositis

Clinical Rheumatology

Author names

Mrudula Joshi [1]

R Naveen [3]

Kshitij Jagtap [2]

Ria Gupta [1]

Vikas Agarwal [3]

Rohit Aggarwal [4]

Ashish Goel [5]

Latika Gupta* [3,6,7,8]

Correspondence to:

Dr. Latika Gupta*

Dept of Rheumatology, Royal Wolverhampton Hospitals NHS Trust, Wolverhampton, WV10 0QP, United Kingdom. ORCID ID: 0000-0003-2753-2990

Email- drlatikagupta@gmail.com

+4401902 307999

Online Resource 1 Definitions of Sources of upload

Source of upload	Definition
Non-medical independent user	An individual who is a consumer and creator of information available on YouTube.
Non-medical media organization	A group engaged in disseminating information to the general public through any medium of mass communication like newspaper, magazine, internet, etc. and not falling into the other categories of sources of upload.
Professional medical society	A group/ regulatory body with a shared scientific interest usually involved in: education and training including Continuing Medical Education (CME), licensing, regulation, ethical issues, setting standards including clinical guidelines, and representing doctors interests [16].
Patient support group	A group of people living with a particular disease who share common experiences and concerns and provide emotional and moral support for one another [17].
