# Supplementary material for: Assessment of quality and reliability of YouTube videos for patient and physician education on inflammatory myositis
Source: Clin Rheumatol. 2023 Feb 9;42(5):1339–49. doi: 10.1007/s10067-023-06522-x (PMC9910767; doi:10.1007/s10067-023-06522-x)
Supplement: Supplementary file 2 — Supplementary file2 (PDF 36 KB) [file 10067_2023_6522_MOESM2_ESM.pdf]

Assessment of Quality and Reliability of YouTube Videos for Patient and Physician Education on Inflammatory Myositis

Clinical Rheumatology

Author names

Mrudula Joshi [1]

R Naveen [3]

Kshitij Jagtap [2]

Ria Gupta [1]

Vikas Agarwal [3]

Rohit Aggarwal [4]

Ashish Goel [5]

Latika Gupta[*] [3,6,7,8]

Correspondence to:

Dr. Latika Gupta[*]

Dept of Rheumatology, Royal Wolverhampton Hospitals NHS Trust, Wolverhampton, WV10 0QP, United Kingdom. ORCID ID: 0000-0003-2753-2990

Email- drlatikagupta@gmail.com

+4401902 307999

Online Resource 2 Analyses by source category

Video characteristics	Hospital (N=62)	Group practice or Physician (N=79)	Nonmedical independent user (N=54)	Nonmedical media organization (N=37)	Professional medical society/Patient support group (N=191)	Pharmaceutical company (N=30)
Usefulness, n (%)						
Useful	53 (85.4)	74 (93.6)	14 (25.9)	27 (72.9)	158 (82.7)	11 (36.6)
Not very useful	1 (1.6)	1 (1.2)	0 (0)	3 (8.1)	3 (1.5)	0 (0)
Patient experience	8 (12.9)	4 (5.0)	40 (74.0)	7 (18.9)	30 (15.7)	19 (63.3)
Content category, n (%)						
Treatment	22 (35.4)	44 (55.7)	4 (7.4)	11 (29.7)	56 (29.3)	6 (20.0)
Etiology	3 (4.8)	15 (18.9)	4 (7.4)	0 (0)	7 (3.6)	1 (3.3)
Diagnosis	20 (32.2)	50 (63.2)	4 (7.4)	12 (32.4)	51 (26.7)	0 (0)
Signs & Symptoms	17 (27.4)	53 (67.0)	6 (11.1)	10 (27.0)	38 (19.9)	1 (3.3)
Ancillary care	1 (1.6)	0 (0)	3 (5.5)	0 (0)	4 (2.1)	0 (0)
Diet	3 (4.8)	0 (0)	0 (0)	0 (0)	5 (2.6)	0 (0)
Physiotherapy	1 (1.6)	0 (0)	0 (0)	0 (0)	21 (10.9)	0 (0)
ADRs	2 (3.2)	0 (0)	0 (0)	0 (0)	5 (2.6)	0 (0)
Physical examination	2 (3.2)	2 (2.5)	1 (1.8)	0 (0)	2 (1.0)	0 (0)
Risk factors	2 (3.2)	1 (1.2)	0 (0)	2 (5.4)	4 (2.1)	0 (0)
Pathogenesis	5 (8.0)	24 (30.3)	0 (0)	1 (2.7)	16 (8.3)	1 (3.3)
Patient experience	8 (12.9)	4 (5.0)	36 (66.6)	6 (16.2)	30 (15.7)	19 (63.3)
Miscellaneous	21 (33.8)	12 (15.1)	11 (20.3)	12 (32.4)	80 (41.8)	2 (6.6)
Intended audience, n(%)						
Anyone/General public	12 (19.3)	5 (6.3)	0 (0)	1 (2.7)	5 (2.6)	1 (3.3)
Specifically for patients	38 (61.2)	19 (24.0)	52 (96.3)	21 (56.7)	164 (85.8)	30 (100)
Healthcare providers including students	50 (80.6)	75 (94.9)	5 (9.2)	27 (72.9)	145 (75.9)	11 (36.6)
Caregivers	7 (11.2)	1 (1.2)	18 (33.3)	7 (18.9)	40 (20.9)	2 (6.6)
Training level, n(%)						
Formal medical training	54 (87.1)	76 (96.2)	3 (5.5)	22 (59.4)	150 (78.5)	11 (36.6)
No formal medical training	8 (12.9)	3 (3.8)	51 (94.4)	15 (40.5)	41 (21.4)	19 (63.3)
Speciality of speaker, n(%)						
Rheumatologist	10 (16.1)	11 (13.9)	0 (0)	3 (8.1)	49 (25.6)	1 (3.3)
Dermatologist	2 (3.2)	0 (0)	0 (0)	0 (0)	5 (2.6)	6 (20)
Neurologist	14 (22.5)	4 (5.0)	1 (1.8)	6 (16.2)	31 (16.2)	0 (0)
GP	6 (9.6)	5 (6.3)	0 (0)	0 (0)	1 (0.5)	0 (0)
Others/Unknown	22 (35.4)	56 (70.8)	2 (3.7)	13 (35.1)	61 (31.9)	4 (13.3)
