# Supplementary material for: Assessment of quality and reliability of YouTube videos for patient and physician education on inflammatory myositis
Source: Clin Rheumatol. 2023 Feb 9;42(5):1339–49. doi: 10.1007/s10067-023-06522-x (PMC9910767; doi:10.1007/s10067-023-06522-x)
Supplement: Supplementary file 3 — Supplementary file3 (PDF 40 KB) [file 10067_2023_6522_MOESM3_ESM.pdf]

Assessment of Quality and Reliability of YouTube Videos for Patient and Physician Education on Inflammatory Myositis

Clinical Rheumatology

Author names

Mrudula Joshi [1]

R Naveen [3]

Kshitij Jagtap [2]

Ria Gupta [1]

Vikas Agarwal [3]

Rohit Aggarwal [4]

Ashish Goel [5]

Latika Gupta* [3,6,7,8]

Correspondence to:

Dr. Latika Gupta*

Dept of Rheumatology, Royal Wolverhampton Hospitals NHS Trust, Wolverhampton, WV10 0QP, United Kingdom. ORCID ID: 0000-0003-2753-2990

Email- drlatikagupta@gmail.com

+4401902 307999

Online Resource 3 Analyses of video characteristics by Score based usefulness (Useful video has mDISCERN >4 or GQS >4 or JAMA >3)

**Video characteristics**	**Useful (N=304)**	**Not very useful (N=41)**	**OR (95%CI)**	**P value**
Audience interaction parameters				
No. of views	921 (296-3146)	579 (160-4408)		0.526
Subscribers	4110 (879-4110)	8330 (468-28200)		0.062
Days since upload	895 (471-1530)	1245 (453-2234)		0.167
Viewing rate	116 (45-307)	76 (32-299)		0.325
Number of likes	12 (4-34)	7 (2-29)		0.130
Number of dislikes	0 (0-1)	0 (0-4.5)		0.139
Interaction index	1 (1-2)	1 (0-2)		0.037
Number of comments	0 (0-3)	1 (0-3)		0.857
Daily viewership	90 [(-457)-2013]	-50 [(-743)-1822]		0.500
Intended audience, n (%)				
Anyone/General public	17 (6)	2 (5)		0.851
Specifically for patients	198 (65)	21 (51)		0.082
Healthcare providers including students	270 (88)	38 (92)		0.453
Caregivers	46 (15)	7 (17)		0.746
Content category, n (%)				
Treatment	120 (39)	19 (46)		0.400
Etiology	25 (8)	5 (12)		0.397
Diagnosis	119 (39)	18 (44)		0.559
Signs & Symptoms	103 (34)	22 (53)	0.4 (0.2-0.8)	0.013
Ancillary care	7 (2)	0 (0)		0.326
Diet	8 (3)	0 (0)		0.293
Physiotherapy	20 (7)	0 (0)		0.091
ADRs	7 (2)	0 (0)		0.326
Physical examination	6 (2)	0 (0)		0.364
Risk factors	7 (2)	2 (5)		0.331
Pathogenesis	40 (13)	7 (17)		0.493

Patient experience	4 (1)	0 (0)		0.460
Miscellaneous	115 (38)	11 (26)		0.170
Sources of upload, n (%)				
Hospital	51 (17)	3 (7)		0.130
Group practice or Physician	53 (17)	22 (54)		<0.001
Nonmedical independent user	12 (4)	2 (5)		0.811
Nonmedical media organization	22 (7)	8 (20)	0.3 (0.13-0.7)	0.012
Professional medical society/Patient support group	159 (52)	2 (5)	21 (5-90)	<0.001
Pharmaceutical company	7 (2)	4 (10)	0.2 (0.06-0.7)	0.019
Training level of the speaker, n (%)				
Formal medical training	284 (93)	30 (73)	11.5 (2.7-48)	<0.001
No formal medical training	20 (7)	11 (27)		<0.001
Speciality of speaker, n(%)				
Rheumatologist	74 (22)	0 (0)		-
Dermatologist	13 (4)	0 (0)		-
Neurologist	55 (16)	1 (13)		0.773
GP	11 (3)	0 (0)		-
Others/Unknown	154 (46)	3 (38)		0.646
Video Quality Metrics				
mDISCERN	4 (3-4)	2 (2-3)		<0.001
GQS	4.5 (4-5)	3 (3-3.5)		<0.001
ADRs Adverse drug reactions, GP General Physician, GQS Global Quality Scale. P<0.05 is significant. Median (IQR) for Scale variables. Chi-square for categorical variables and Mann Whitney U test for scale variables				
